# The use of real time strain endometrial elastosonography plus endometrial thickness and vascularization flow index to predict endometrial receptivity in IVF treatments: a pilot study

**DOI:** 10.1186/s12880-023-01071-w

**Published:** 2023-09-15

**Authors:** Antonio Stanziano, Francesco Paolo Bianchi, Anna Maria Caringella, Clementina Cantatore, Antonio D’Amato, Angela Vitti, Anna Cortone, Amerigo Vitagliano, Giuseppe D’Amato

**Affiliations:** 1 Department of Advanced Reproductive Risk Management and High-Risk Pregnancies, ASL Bari, Reproductive and IVF Unit, PTA Conversano, Conversano, BA Italy; 2 Department of Interdisciplinary Medicine, Aldo Moro, Bari, Italy; 3 Gynecology and Obstetrics Clinic, University of Bari, Aldo Moro, Bari, Italy

**Keywords:** Endometrial thickness, Strain elastosonography, Endometrial vascularity, Endometrial receptivity, IVF cycles, Pregnancy rate.

## Abstract

**Background:**

The usefulness of endometrium strain elastosonography (SE) for the evaluation of endometrial receptivity in women undergoing in vitro fertilization (IVF) remains controversial. The objective of this prospective, observational study was to evaluate the correlation between endometrial thickness (EMT) and its related strain (ESR) on the day of ovulation triggering (hCG-d) and in vitro fertilization outcomes. Additionally, 3D Power Doppler vascular indices (3DPDVI) were also analysed.

**Methods:**

We included all the patients undergoing fresh IVF-single blastocyst transfer cycle from January 2021 to August 2021 at our center. On hCG-d, after B-mode scanning was completed to measure the EMT, the mode was changed to elastosonography to evaluate the ESR (ratio between endometrial tissue and the myometrium below). At the end of examination, the Endometrial Volume (EV) and 3DPDVI (vascularization index [VI], flow index [FI] and vascularization flow index [VFI]), were assessed. Statistical analysis was completed using STATA MP16 software.

**Results:**

A total number of 57 women were included. Based on the EMT on hCG-d, women were divided into two groups, Group 1: <7 mm and Group 2 ≥ 7 mm. Women with EMT < 7 mm had a significantly higher ESR (p = 0.004) and lower pregnancy rate (p = 0.04). Additionally, low ESR values were correlated with high VFI values (rho = -0.8; 95% CI = -0.9- -0.6; p < 0.0001) and EMT ≥ 7 mm could be predicted by low ESR (OR = 0.01; 95% CI = 0.01–0.30; p = 0.008, area under the ROC curve: 0.70). After all, in multiple logistic regression analysis, low values of ESR (*p* = 0.050) and high values of EMT (*p* = 0.051) on hCG-d had borderline statistical effects on pregnancy rate.

**Conclusions:**

The ESR may be useful to improve the ultrasound evaluation of the endometrial quality in infertile women candidates to IVF/ICS. Given the small sample size of our study, the usefulness of strain elastosonography in this patients, needs further investigation.

**Supplementary Information:**

The online version contains supplementary material available at 10.1186/s12880-023-01071-w.

## Background

The evaluation of endometrial quality is crucial in Assisted Reproductive Technology (ART), where the selected embryos should ideally be transferred to a receptive *niche* [1, 2]. In this respect, it is accepted that the embryo, the endometrium and the “cross-talk” between these two actors accounts for one-third of implantation failure, respectively [3].

Transvaginal Ultrasonography (TVU) is the gold standard technique for evaluating endometrial receptivity (ER) due to its low cost, good patient tolerance, and low invasiveness [4]. In the majority of published studies, monitoring of ER using ultrasound takes into account the echogenic pattern (type A: triple layer, characterized by a hypoechoic endometrium with a central line, type B: isoechoic endometrium with poorly defined outer walls and central echogenic line and type C: homogeneous hyperechoic morphology) [5] and thickness of the endometrium [6, 7].

A *“thin”* endometrium has been reported in 5% of women < 40 years old and 25% of women aged 41–45 years old, and it has also been related to poor pregnancy outcome [8]. Differently, in other studies, the incidence of Endometrial Thickness (EMT) < 7 mm on the day of hCG administration is lower, ranging between 1% and 2.5% of IVF patients [9, 10].

Notably, during controlled ovarian stimulation the endometrium undergoes morphologic changes due to a rise of serum estradiol produced by developing follicles. This process leads to an increased vascularity of the endometrium, with angiogenic vessels developing along the elongation of the glandular crypts of the functional layer [11–13].

Endometrial vascularity can be evaluated by using power Doppler technology. Some studies showed that the characteristics of endometrial and subendometrial blood flows at the time of ovulation triggering may influence the IVF outcome [14, 15], although solid evidence is still needed [16, 17]. The advent of TVU with 2D and 3D Power Doppler Angiography (3D US PDA) has provided an instrument to measure endometrial and subendometrial blood flows by using the following indices: Vascularization Index (VI), Flow Index (FI), Vascularization Flow Index (VFI), [18, 19] and Endometrial Volume (EV) [20]. Their value in predicting ART cycle outcome has been increasingly considered in recent years, with conflicting results [21–25].

Strain Elastosonography (SE) is a new US technique evaluating tissue elasticity. This technique is performed using the transducer analyzing radiofrequency echo data during manual freehand compressions of the tissue, giving a picture of the distribution of deformation or stiffness in an organ in response to an applied stress. Previously, this technique was mainly used for differential diagnosis of masses in breast and thyroid tissues [26, 27]. Recently, different studies have reported interesting new data for clinical SE applications in gynecology [28–31], obstetrics [32–35] and ARTs [36–38]. Specifically, endometrial SE may reflect the endocrine, paracrine and autocrine interactions that take place in the endometrium during an ART cycle.

The aim of this cross-sectional study is to evaluate the possible correlation between EMT and its equivalent strain condition on hCG-d in women undergoing their first in vitro fertilization treatment and fresh embryo transfer. Moreover, we compared the endometrial 3D US PDA with the equivalent strain parameters.

## Methods

This prospective, observational study was conducted at “Reproductive and IVF Unit” of Conversano (ASL BA, Bari, Italy) from January 2021 to August 2021. We recruited all those women who were candidates to IVF and satisfied the following entry criteria (57 women): Age < 40 years; antral follicle count [AFC] ≥ 5; anti-Müllerian hormone [AMH] ≥ 1.2ng/ml; morphologically normal uterus (no intrauterine adhesions, fibroids, adenomyosis and polyps) confirmed by standard and three-dimensional (3D) transvaginal ultrasound and hysteroscopy; single, fresh blastocyst transfer (SET); 4) no history of uterine or endometrial surgery.

Exclusion criteria were plasma E_2_ > 3000 pg/ml, progesterone (P) level > 1.5ng/ml, EMT > 14 mm or < 5 mm and an endometrial pattern B or C on the day of hCG priming. These patients were excluded because the fresh transfer was not performed. Women with retroverted uterus were also excluded, due to the difficulty of obtaining adequate endometrial findings of SE. Informed consent was obtained from all patients and the study was approved by the Institutional Ethical Committee (IEC) on November 27, 2019 (IEC 6167/2019). The study was conducted according to the provisions of the Declaration of Helsinki.

All women received the same GnRH antagonist (GnRH-a) stimulation protocol. 150 IU/day of Follitropin alfa biosimilar (FSH-r) (Bemfola®, Finox Biotech, Switzerland) was started on the second day of the menstrual cycle. The injection of GnRH antagonist (Orgalutran® 0,25 mg, MSD, Italy) was administered once the diameter of the dominant follicle reached 14 mm and was continued up to the trigger day. The dose of FSH-r was adjusted according to the follicle response. As soon as the diameter of the dominant follicle was greater than 20 mm or when at least three follicles reached 18 mm, ovulation induction was co-triggered with 10.000 IU hCG (Gonasi® HP, Ibsa, Italy) 34–36 h prior to egg retrieval [39]. Ultrasonographic TVU examination including endometrial thickness, strain elastosonography and 3D measurements were performed by one trained and expert observer (A.S.) with more than 10 years of gynecologic ultrasound experience. The luteal phase was supported by vaginal progesterone, 200 mg three times a day (Progeffik® 200 mg Effik, Italy). A single blastocyst was transferred five days after oocyte retrieval. Clinical pregnancy was defined as the presence of a visible fetal heartbeat under transvaginal ultrasonography, 4 weeks after the embryo transfer.

### Ultrasound Equipment

**Fig. 1:**
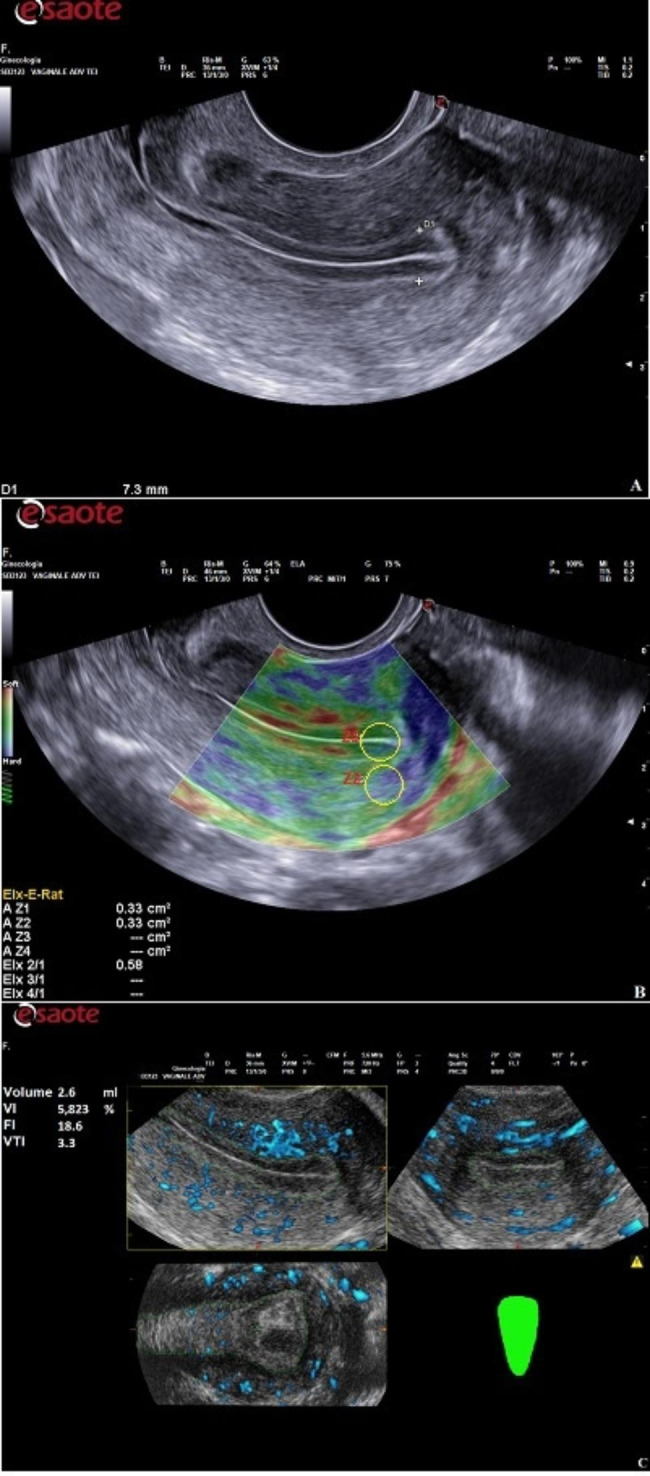
The ultrasound examination protocol: **(A)** B mode US shows a normal anteverted uterus with endometrial thickness of 7.3 mm. **(B)** Endometrial elastosonography and strain ratio (Z2/Z1) calculation in a sagittal plane: endometrium (Z1), adjacent myometrium (Z2). The strain rate (SR) is calculated automatically by the software. SR = 0.58. **(C)** Endometrial Volume (EV) and 3D Power Doppler vascularity indices are calculated automatically after a manually reconstruction using the Virtual Organ Computer-aided Analysis (VOCAL) software (XVRA ™, Esaote, Italy). Endometrial volume = 2.6ml, VI = 5.823%, FI = 18.6, VFI = 3.3

A digital ultrasound (US) scanner (MyLab™ XPro80, Esaote, Italy), equipped with a volumetric multifrequency endovaginal probe (SB3123, 3–12 MHz), was used. After placing the endovaginal probe in the anterior fornix of the vagina, a clear view of the endometrium was obtained. The sagittal view of the uterus was first viewed in B-mode modality: in this plane, the endometrial thickness was measured as the maximum distance between the 2 interfaces of endometrial– myometrial junction (Fig. 1A). Endometrial patterns were classified according to the morphology of the endometrium as: pattern A (triple-line type characterized by a hypoechoic endometrium with well-defined hyperechoic outer walls and a central echogenic line); pattern B (isoechoic endometrium with poorly defined outer walls and central echogenic line); pattern C (homogeneous hyperechoic endometrium). The values for endometrial thickness and the pattern were recorded for later analysis. After B-mode scanning was completed, the US modality changed to elastosonography, to evaluate the endometrial strain. The acquisition was performed in the sagittal plane of the uterus, with a clear view of the endometrium and adjacent myometrium. ElaXto ™ strain technology was then activated and a dual-mode screen was used in order to display the conventional B-mode on the left and the strain regions of interest (ROI) elastogram on the right. Endometrial strain was performed using a freehand technique, by applying with the probe light and repetitive cycles of perpendicular pressure (tissue compression-relaxation) through rhythmic movements each lasting about 1 s on the mid-sagittal plane, as suggested by previous studies [38, 40]. The ElaXto Spring Tool displayed on the screen was a real-time feedback indicator of correct performance of the strain image acquisition. The procedure was then repeated, to acquire two raw datasets for each patient. When the indicator turned to green, the image was frozen for measurements of strain values and elastographic images. During the SE evaluation, an elastogram with a colour scale palette, ranging from red to blue and representing the degree of tissue elasticity, was displayed on the screen; red signals represented tissues with high elasticity and blue signals represented tissues with low elasticity (Fig. 1B). Elastogram ROI dimension and position could be modified by the operator, to achieve the best real-time acquisition. Consequently, the ElaXto Ratio tool was selected in the elastographic measurement tab. The reference area for the ratio was the endometrial tissue, with a distance between the fundal endometrial surface of 1.5-2 cm (Z1) and the myometrium below (Z2). In order to include the endometrium, two circles (Z1 and Z2) ranging from 5 to 9 mm were placed on the ROI elastogram. The Strain Rate (SR) was calculated automatically by the software, indicating the strain ratio, in particular, a higher SR indicated that the tissue was stiffer. Three measurements (using ELX-E-RAT, i.e. ElaXto Ellipse Ratio measure) were performed for each patient, to determine mean SR values, and these values were recorded. The ElaXto software processed the elastographic images. We will refer to these values in the following sections by using the term Endometrial Strain Rate (ESR).

At the end of examination, the Endometrial Volume (EV) and 3D Power Doppler vascularity indices were assessed. The settings for this study were as follows: color gain: 50%; normal quality of colour; PRF: 470 Hz; B-mode focal depth: 3 cm; persistence: 4; central frequency: mid; smooth: mid; line density: 3; PD map: P0A; frequency: 6.3 MHz; flow resistance: 3; quality: high; field of view of 22°-241° and radius of curvature of 10 mm. A new angiosonographic volume was acquired if Doppler artifacts were present, due to respiratory or intestinal movement. These US volumes were stored and post-processed by the same investigator on a personal computer (Fig. 1C).

For each three-dimensional acquisition, the endometrial volume was manually reconstructed in the coronal plane using the Virtual Organ Computer-aided Analysis (VOCAL) software (XVRA ™, Esaote, Italy) with a sweeping angle of 30°. XVRA automatically calculates the EV and three angiosonographic Power Doppler indexes: vascularization index (VI), the ratio of colour voxels to all voxels in the region of interest expressed as percentage, Flow Index (FI) which relates the intensity of the color voxel and Vascularization Flow Index (VFI), which relates the VI and FI to each other; these three indexes could be clinically related with the number of vessels, blood flow and endometrial perfusion, respectively [41].

### Statistical analysis

Endometrial thickness was first considered as a categorical variable and, therefore, women were divided into two EMT groups, <7 mm (Group 1) vs. ≥7 mm (Group 2). Data from the compiled forms were entered into a database generated using Excel and analyzed using STATA MP16 software. Continuous variables were reported as mean ± standard deviation and range, and categorical variables as proportions. Skewness and kurtosis tests were conducted to evaluate the normality of the continuous variables; in case of a non-normal distribution, a normalization model was established. Student’s t-test for independent data (parametric) and the Wilcoxon rank sum test (non-parametric) were used to compare continuous variables between groups. Chi-squared and Fisher’s exact tests were used to compare proportions. Spearman’s rank correlation test was used to assess the strength and direction of the monotonic relationship between EMT, ESR and VFI; assessing the correlation of one variable at a time; the rho value was indicated together with the 95% CI the 95% confidence interval (95%CI). To assess the strength of the correlation the below reported scale of absolute values of rho was used:

- Absolute value rho ≤ 0.2: very weak correlation.
- Absolute value 0.2 < rho ≤ 0.4: weak correlation.
- Absolute value 0.4 < rho ≤ 0.6: moderate correlation.
- Absolute value 0.6 < rho ≤ 0.8: strong correlation.
- Absolute value rho > 0.8: very strong correlation.

Additionally, the statistical significance of the correlation was assessed using the significance test for the Spearman’s correlation coefficient.

As a way to assess the determinants of pregnancy (YES/NO), a multivariate logistic regression model was used in which pregnancy was the outcome and SE, ESR, VFI and the patient age were the determinants. The adjusted odds ratio (aOR) was calculated together with the 95%CI. Univariate logistic regression was used to evaluate the association between EMT (> 7 mm vs. ≤7 mm) and ESR and VFI; the Odds Ratio (OR) values were indicated together with the 95% CI. Subsequently, the ROC curves were used to evaluate the predictivity of VFR and ESR, with the indication of the area under the curve. The Youden Index was used to evaluate the cut-off of the ESR and VFI variables, but for the ESR variable the test did not identify a unique value, probably due to the small sample size. For all tests, a two-sided p-value < 0.05 was considered to indicate statistical significance.

## Results

A total number of 57 women were included in the study. 42 women (73.7%) had an EMT ≥ 7 mm, and 15 (26.3%) had EMT < 7 mm. The two groups did not differ in age, basal FSH, LH, E2, AMH levels, AFC, BMI, number of days of stimulation and antagonist administration, E2 and P concentration on the hCG injection day (p > 0.05) (Table 1).

No differences were found between groups in terms of VI, FI, and VFI. Conversely, patients with EMT < 7 mm had a significantly higher ESR (t = 2.98; p = 0.004) and a significantly lower VE (t = 3.95; p < 0.001) on the day of hCG priming compared to those with EMT ≥ 7 mm. Moreover, the clinical pregnancy rates was statistically lower in women with endometrial thickness < 7 mm versus ≥ 7 mm (13.3% versus 42.9%; *X*^2^ = 4.22; p = 0.04) (Tables 2 and 3). The first graph shows that there was no statistically significant correlation between EMT and ESR (rho = -0.2; 95% CI = -0.4–0.1; p = 0.202;) likewise between EMT and VFI (rho = 0.03; 95% CI = -0.2- 0.3; p = 0.841; graphic 2), while a statistically significant inverse correlation was observed between ESR and VFI (rho = -0.8; 95% CI = -0.9-0.6; p < 0.0001; graphic 3). According to the univariate logistic regression model, the occurrence of EMT ≥ 7 mm could be predicted by low ESR (OR = 0.01; 95% CI = 0.01–0.30; p = 0.008), the area under the ROC curve was 0.70 (95%CI = 0.53–0.88); (graphic 4). On the contrary, the graphic 5 shows no statistically significant associations between EMT and VFI (OR = 1.4; 95% CI = 0.7–3.1; p = 0.376), the area under the ROC curve was 0.56 (95%CI = 0.38–0.71) and the optimal cut-off value identified by the Youden Index was 0.25. The multiple logistic regression analysis reported in Table 3 showed that the occurrence of clinical pregnancy after IVF could be predicted by low values of ESR (*p* = 0.051) and high values of EMT (*p* = 0.050), with borderline statistical significance. Otherwise, we observed that women’s age and endometrial VFI were not correlated with pregnancy rate (*p* = 0.318 and *p* = 0.197, respectively). The proportion of positive pregnancy tests predictable by the model was approximately 44.1%.

A post-hoc power analysis was performed based on the values of ESR at the day of hCG; an alpha error probability of 0.05 was set and a power (1-β error) of 90% was estimated. This analysis was performed by G*Power 3.1 software.

**Table 1:** Characteristics of the studied population and ovarian stimulation parameters according to the endometrial thickness

	Group 1	Group 2	Total	Test	p-value (0.05)
**Variable**	**EMT < 7 mm** **(n = 15)**	**EMT ≥ 7 mm** **(n = 42)**	**(n = 57)**		Not applicable
Age (years)	33.7±1.5 (30–35)	34.0±1.7 (29–39)	33.9±1.6 (29–39)	z = 0.67	0.501
Basal FSH (mIU/ml)	7.0±2.0 (2.1–9.7)	7.1±1.8 (1.9–10.0)	7.0±1.9 (1.9–10.0)	t = 0.21	0.836
Basal LH (mIU/ml)	5.2±2.0 (1.4-9.0)	5.1±1.8 (1.5-9.0)	5.1±1.8 (1.4-9.0)	t = 0.21	0.834
Basal E_2_(pg/ml)	46.6±20.9 (13–78)	52.7±26.9 (16–130)	51.1±25.4 (13–130)	t = 0.81	0.428
AMH ng/ml	2.3±1.5 (1.2–6.3)	2.7±2.2 (1.2–10.0)	2.6±2.0 (1.2–10.0)	t = 0.69	0.497
AFC n.	9.4±4.5 (5–19)	10.6±4.1 (5–20)	10.3±4.2 (5–20)	t = 0.94	0.351
BMI (kg/m^2^)	22.3±1.5 (18.9–24.8)	22.4±1.2 (19.8–24.4)	22.3±1.4 (18.9–24.8)	t = 0.64	0.47
Stimulation duration (days)	10.5±1.0 (9–13)	10.8±0.9 (9–13)	10.7±0.9 (9–13)	t = 1.17	0.245
Antagonist administration duration (days)	4.1±0.7 (3–5)	3.8±0.7 (3–5)	3.9±0.7 (3–5)	t = 1.15	0.253
E_2_ at hCG-d (pg/ml)	1371.4±671.8 (471–2368)	1400.4±629.8 (425–2718)	1392.8±635.1 (425–2718)	t = 0.15	0.881
P at hCG-d (ng/ml)	0.91±0.23 (0.3–1.5)	0.89±0.10 (0.3–1.5)	0.9±0.09 (0.3–1.5)	t = 1.38	0.175

**Table 2:** Ultrasonographic findings of endometrium on the day of hCG and the pregnancy rate according to the endometrial thickness

	Group 1	Group 2	Total	Test	p-value (0.05)
	EMT < 7 mm (n = 15)	EMT ≥ 7 mm (n = 42)	(n = 57)		
ESR at hCG-d	0.6±0.2 (0.2–0.9)	0.4±0.2 (0.2–0.9)	0.5±0.2 (0.2–0.9)	t = 2.98	0.004
EV (ml) at hCG-d	1.6±0.6 (0.7–3.1)	2.7±1.0 (1.4–5.9)	2.4±1.0 (0.7–5.9)	t = 3.95	<0.001
VI% at hCG-d	4.8±3.2 (0.7–11.0)	5.6±3.5 (1.43–13.6)	5.4±3.4 (0.7–13.6)	t = 0.70	0.485
FI at hCG-d	20.3±5.6 (2.4–25.6)	22.4±2.5 (18.7–29.0)	21.9±3.6 (2.4–29.0)	t = 1.57	0.121
VFI at hCG-d	1.1±0.7 (0.1–2.2)	1.3±0.9 (0.2–3.9)	1.2±0.8 (0.1–3.9)	t = 0.88	0.381
Pregnancy rate	2/15 (13.3%)	18/42 (42.9%)	20/57 (35%)	*X*^2^ = 4.22	0.040

**Table 3:** Multivariate analysis after stepwise downward logistic regression

Parameters	aOR	95%CI	p-value
EMT	1.5	1.0-2.4	0.051
ESR	2.1*10^-4	4.6*10^-8–0.99	0.050
VFI	2.9	0.6–15.0	0.197
AGE	1.3	0.8–2.1	0.318

## Discussion

To the best of the team knowledge, this is the first study evaluating the relationship between endometrial elasticity and EMT in infertile patients undergoing fresh IVF cycles. Elastosonography is a recent US technology with a growing range of applications in medicine. Specifically, there are two modalities of elastosonographic imaging: SE and Shear Wave Elastosonography (SWE). These two techniques differ in the imaging method and the type of force applied for tissue compression. Briefly, the SE procedure is a static study that examines the distortion of the tissue when a pressure is applied. The SWE is a dynamic study that determines tissue elasticity by applying an Acoustic Radiation Force Impulse (ARFI), generated by the US device, and measuring the velocity of the shear waves propagating within the tissue. Notably, the static method is widely dependent on the sonographer’s experience and is considered less reproducible than the dynamic one [42, 43]. Only one study compared the diagnostic accuracy of SE and SWE at the same time; results were comparable between these two methods. Conversely, a recent meta-analysis pooled individual studies of SE and SWE and found that SWE had higher accuracy compared to SE [44]. Therefore, there is still no clear consensus on the best technique between SWE and SE [45], and the latter one remains a valid technique [46–50].

The diagnostic performance of SE depends both on the applied force and on the diameter and location of the ROI. The strain ratio has been commonly used in clinical practice, and corresponds to an approximation of the Young’s modulus ratio between two tissue regions, assuming applied force is equivalent across these regions.

All these factors should be taken into account when this technique is adopted, as well as when its results are evaluated. Swierkowski-Blanchard et al. [36] examined the endometrial elasticity by SE in women undergoing IUI. In their study, the region of interest was the subendometrial myometrium and the control was the endometrium, reporting higher elasticity index in pregnant women (2.4 ± 1.3) compared to non-pregnant (1.5 ± 0.7). Instead, recently, Cihan Kabukçu et al. assessed the ESR comparing the endometrium (region of interest) with an area of the parametrial tissue with highest elastic color scale (control) and they concluded that ESR was not a useful marker for pregnancy prediction in unexplained infertility cases undergoing IUI [38]. In their study, the reference area for the ratio was the endometrial tissue (Z1) and the myometrium below (Z2), with a distance between the fundal endometrial surface of 1.5-2 cm: the area of embryo implantation. Due to the scarcity of available data and the requirement for a large number of examined patients, it is still not possible to make predictive estimates of ART success based on variables such as endometrial pattern, thickness, or 3D US PDA parameters [10, 25, 51].

Our group decided to evaluate endometrial patterns and elastosonography on the trigger day because the majority of studies concluded that the endometrial characteristics, as assessed on the day of hCG administration, can predict the ongoing pregnancy rate. Parameters used in these studies included endometrial thickness, endometrial pattern, endometrial and sub-endometrial blood flow [2, 8]. Therefore, the aim of the present study was to evaluate the possible correlation between EMT and its equivalent strain condition on hCG Day.

Thin endometrium on ultrasound during ovarian hyperstimulation is associated with poor success rate not only after IVF but also in unstimulated menstrual cycle. As shown in Kasius’s meta-analysis the probability of conceiving with EMT ≤ 7 mm was significantly lower when compared with EMT > 7 mm [10]. The EMT cut off ≥ 7 mm in most studies is the value above which there is an improvement in pregnancy rates. Furthermore, endometrium < 7 mm is also associated with a lower clinical pregnancy rate in IVF cycles [11]. Thin endometrium (< 7 mm) was considered as a negative prognostic factor for achieving pregnancy also in women without ovarian stimulation [52, 53].

As shown in the present study, patients included in the analysis had balanced demographic data and ovarian responses.

The EMT ≥ 7 mm group had lower ESR compared with the EMT < 7 mm group (p 0.004). Furthermore, in patients with EMT < 7 mm, VE is significantly lower. The VI, FI and VFI parameters did not show significant difference between the two groups. In line with previous studies, the pregnancy rate in the EMT < 7 mm group was significantly lower (13.3%) than the EMT ≥ 7 mm group (42.9%) (*p*. 0.040) [11]. Although the pregnancy rate does not represent the aim of the study, we reported the occurrence of clinical pregnancy after IVF could be predicted by low values of ESR (*p* = 0.051) and high values of EMT (*p* = 0.050). It is also important to analyze EMT, ESR and VFI results: the relationship between EMT and ESR, EMT and VFI, ESR and VFI was evaluated, and it was possible to observe that a significant correlation exists only between ESR and VFI (p < 0.0001). In addition to this, the occurrence of EMT ≥ 7 mm could be predicted by low ESR (area under the ROC curve = 0.70). These results can be interpreted according to the main histological knowledge of the endometrium. Indeed, in the endometrium there are two primary layers: the upper is functionalis and lower basalis. Hess and his research team also found several cell types, including glandular epithelium, luminal epithelium, stromal fibroblasts, Collagen I, vascular smooth muscle, blood vessels and an array of immune cells and stem or progenitor cells [54]. The inner surface of the endometrium is lined with a single layer of pseudostratified epithelium: the luminal epithelium. Invaginations of the epithelium form coiled and slightly branched glands that extend from the lumen to the inner circular layer of the myometrium. Stromal cells and an extracellular matrix support both luminal and glandular epithelia [55]. The functional layer has a vascular capillary structure, in contrast to the larger spiral arteries in the basal layer. Casper et al. reported in the endometrium, when the thickness measured by ultrasound is < 7 mm, the functional layer is thin, or absent [56]. Miwa et al. demonstrated that the thin endometrial layer is characterized by glandular epithelium poor growth and low VEGF protein expression related to high blood flow impedance of radial arteries [57]. Other investigators have focused their attention on the pathological changes in the endometrium, linked to changes in its rigidity, due to an increased proportion of collagen fibers in the endometrium and a reduction of the endometrial glandular epithelium. Overall, these factors lead to the increase in the endometrial stiffness [58, 59].

Interestingly, we did not observe significant differences between the groups with endometrial thickness (EMT) < 7 mm and ≥ 7 mm in terms of VI, FI, and VFI parameters. Although these results may appear contradictory to the significant correlation between ESR and VFI, further clarification is necessary to explore possible explanations. Thin endometrium is a pathologic entity that is still poorly defined and can be attributed to various underlying physiopathological factors, including inflammatory conditions, idiopathic causes, previous surgeries, fibrotic sequelae of prior infections, spontaneous aging of basal cells, and receptor insensitivity to estrogen [60–68]. Since histological evaluation of the endometrium was not feasible in our study (as patients underwent embryo transfer), it is possible to hypothesize that our patients had heterogeneous causes of thin endometrium, with variable presence of the functional layer from patient to patient. This factor, along with the low number of patients examined, may explain why a clear profile of stiffness and vascularization could not be identified in the presence of different endometrial thicknesses. However, it should be noted that patients with thin endometrium are relatively rare (5% or less of the population undergoing IVF), making it extremely challenging to achieve a large number of patients for study [8].

Our study suffers from some limitations. An important methodological limitation was the inability to assess endometrial strain directly but rather through a surrogate variable (ESR). This inevitably introduces a bias that needs to be taken into consideration when evaluating our findings by the reader. Additionally, all Angiosonographic Power Doppler indexes applied in our study (vascularization, flow, and vascularization/flow) do not provide an exact estimation of endometrial blood flow, but rather approximate estimations, which may introduce interpretative bias.

A further limitation of the present study was the unavailability of intra and interobserver reliability in the assessment of SE: to minimize its impact, three measurements were performed for each patient by the same expert operator, to determine mean SR values. Moreover, the small sample size was a further point of weakness of the study. In this respect, multivariate regression analysis showed a trend towards higher pregnancy rate with decreasing ESR, but statistical significance was not reached (p = 0.05). On one hand, this result certainly limits drawing any firm conclusion about the value of ESR as an independent predictor of the IVF outcome. On the other hand, multivariate regression analyses suffer from small sample sizes, as evidenced by the lack of statistical correlation between other well-established predictors of IVF outcome (age, EMT) and pregnancy rate in our study. However, the fact that ESR and ET had a similar impact on the IVF outcome can be considered as a promising finding that warrants future confirmation on a larger population.

## Conclusions

Our study found that ESR and EMT had similar value in the prediction of the outcome of fresh blastocyst transfer. Even if our findings may be promising, the small sample size of our study limits drawing any firm conclusions about the usefulness of real time strain elastosonography as a tool for the evaluation of endometrial receptivity. Therefore, further well-conducted, prospective studies on larger populations are needed.

### Electronic supplementary material

Below is the link to the electronic supplementary material.

Graphic 1

Graphic 2

Graphic 3

Graphic 4

Graphic 5

Legends supplementary graphics

## Data Availability

The datasets used and/or analyzed during the current study are available and can requested to the corresponding author on reasonable request.

## Abbreviations

3DPDVI: 3D Power Doppler vascular indices
3D US PDA: 3D Power doppler angiography
AMH: anti-Müllerian hormone
AFC: Antral follicle count
ARFI: Acoustic radiation force impulse
ART: Assisted reproductive technology
ELX-E-RAT: ElaXto Ellipse Ratio measure
EMT: Endometrial thickness
ER: Endometrial receptivity
ESR: Endometrial strain rate
ET: Embryo transfer
EV: Endometrial volume
FI: Flow index
IVF: In vitro fertilization
hCG-d: human Chorionic Gonadotropin administration day
ROI: Region(s) of interest
SE: Strain elastosonography
SET: Single embryo transferred
SWE: Shear wave elastosonography
TVU: Transvaginal ultrasonography
UE: Ultrasound elastosonography
US: Digital ultrasound
VFI: Vascularization flow index
VI: Vascularization index
VOCAL: Virtual organ computer-aided analysis
