# Supplementary figures and images for: The use of real time strain endometrial elastosonography plus endometrial thickness and vascularization flow index to predict endometrial receptivity in IVF treatments: a pilot study
Source: BMC Med Imaging. 2023 Sep 15;23:130. doi: 10.1186/s12880-023-01071-w (PMC10503140; doi:10.1186/s12880-023-01071-w)

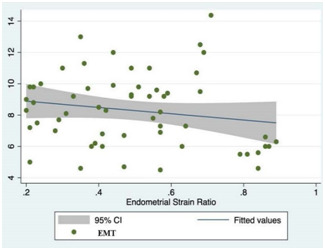

Supplement: Supplementary file 1 — Graphic 1 [file 12880_2023_1071_MOESM1_ESM.jpeg]

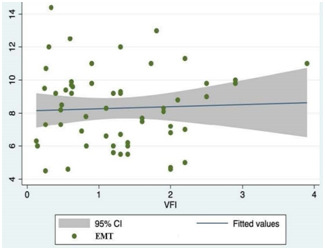

Supplement: Supplementary file 2 — Graphic 2 [file 12880_2023_1071_MOESM2_ESM.jpeg]

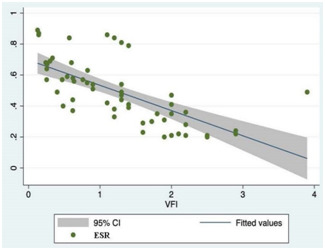

Supplement: Supplementary file 3 — Graphic 3 [file 12880_2023_1071_MOESM3_ESM.jpeg]

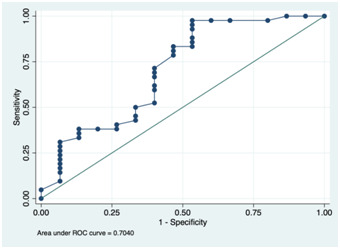

Supplement: Supplementary file 4 — Graphic 4 [file 12880_2023_1071_MOESM4_ESM.jpeg]

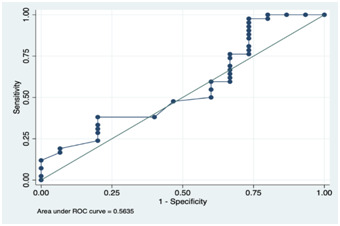

Supplement: Supplementary file 5 — Graphic 5 [file 12880_2023_1071_MOESM5_ESM.jpeg]
