# Supplementary material for: The use of real time strain endometrial elastosonography plus endometrial thickness and vascularization flow index to predict endometrial receptivity in IVF treatments: a pilot study
Source: BMC Med Imaging. 2023 Sep 15;23:130. doi: 10.1186/s12880-023-01071-w (PMC10503140; doi:10.1186/s12880-023-01071-w)
Supplement: Supplementary file 6 — Legends supplementary graphics [file 12880_2023_1071_MOESM6_ESM.docx]

**Legends supplementary graphics**

**Graphic 1:** Correlation between EMT and ESR (p = 0.202)

**Graphic 2:** Correlation between EMT and VFI (p = 0.84)

**Graphic 3:** Correlation between ESR and VFI (p <0.0001)

**Graphic 4:** ROC curve of ESR for predicting EMT

**Graphic 5:** ROC curve of VFI for predicting EMT
